# Conserving Semantic Unit Information and Simplifying Syntactic Constituents to Improve Implicit Discourse Relation Recognition

**DOI:** 10.3390/e25091294

**Published:** 2023-09-04

**Authors:** Zhongyang Fang, Yue Cong, Yuhan Chai, Chengliang Gao, Ximing Chen, Jing Qiu

**Affiliations:** The Cyberspace Institute of Advanced Technology, Guangzhou University, Guangzhou 510006, China; 1111906003@e.gzhu.edu.cn (Z.F.); 1111906002@e.gzhu.edu.cn (Y.C.); chaiyuhan@e.gzhu.edu.cn (Y.C.); 1112106002@e.gzhu.edu.cn (C.G.); 2112019039@e.gzhu.edu.cn (X.C.)

**Keywords:** implicit discourse relation recognition, shallow discourse parsing, relation extraction, phrase extraction

## Abstract

Implicit discourse relation recognition (IDRR) has long been considered a challenging problem in shallow discourse parsing. The absence of connectives makes such relations implicit and requires much more effort to understand the semantics of the text. Thus, it is important to preserve the semantic completeness before any attempt to predict the discourse relation. However, word level embedding, widely used in existing works, may lead to a loss of semantics by splitting some phrases that should be treated as complete semantic units. In this article, we proposed three methods to segment a sentence into complete semantic units: a corpus-based method to serve as the baseline, a constituent parsing tree-based method, and a dependency parsing tree-based method to provide a more flexible and automatic way to divide the sentence. The segmented sentence will then be embedded at the level of semantic units so the embeddings could be fed into the IDRR networks and play the same role as word embeddings. We implemented our methods into one of the recent IDRR models to compare the performance with the original version using word level embeddings. Results show that proper embedding level better conserves the semantic information in the sentence and helps to enhance the performance of IDRR models.

## 1. Introduction

Discourse relation recognition (DRR) aims at identifying the semantic relation of two arguments in a discourse. This task can be further categorized into explicit discourse relation recognition (EDRR) and implicit discourse relation recognition (IDRR). EDRR refers to the case when an explicit connective, such as because or but, exists between two text segments, while IDRR deals with the discourse without such connectives. The studies on EDRR have shown a satisfying result [1], as a connective is usually a clue strong enough to predict the relation. As for IDRR, the absence of explicit connectives requires further understanding of the involved text and lead to much more difficulty in recognizing the relation. The results of IDRR studies are still far from satisfactory [2].

IDRR has long been one of the most challenging tasks in shallow discourse parsing as well as in natural language processing [3,4]. As the relation between two spans of text can provide rich semantic information, IDRR is also a fundamental step for many downstream tasks, such as text summarization [5,6,7], question answering [8,9,10], and eventually some IoT or malware detection tasks that need to predict relations between things [11,12].

The methods of studying IDRR can be roughly divided into the machine learning way and the deep learning way. Machine learning-based methods use linguistics features to capture semantic information inside an argument pair. Common linguistic features include lexical features [13,14], contextual features [15,16], syntactic features [17,18], and implicit connectives [19,20]. Such features will then be encoded into numerical vectors and fed into a machine learning classifier to predict the final relation. While the machine learning way relies on some human-designed patterns to extract the required linguistic features, deep learning-based methods, instead, use neural networks to automatically capture semantic information.

Deep learning has been successfully used in various different domains [21,22]. For IDRR, deep learning-based methods outperform the machine learning way in existing works and achieve an F1 score of over 60% [23]. However, deep learning-based methods face the same potential problem, that the rich semantic information of the phrases in natural language may not be fully exploited, while phrasal information is helpful for the IDRR problem. Existing studies usually take word or subword level tokens as network inputs, which would inevitably lead to a deterioration of the phrase meaning itself [1].

Phrases usually constitute a complete semantic unit by taking the job of a certain constituent or word class. For example, phrasal verbs refer to phrases that act as a predicate and prepositional phrases are some locutions with the same role as a preposition. Isolated understanding of the words in a phrase would bring a loss of semantic information. However, it is not to say that only phrases can be a semantic unit. The discrimination of a semantic unit depends on the function the word(s) perform(s) in a sentence. For example, the word takes stands for a complete semantic unit in the sentence, Mike takes a cup of milk every day. However, for the case, nobody took up for Mike in this election, the word took has to be combined with up and for to make the phrasal verb, take up for, and convey the meaning of support.

Our work is conceived out of two motivations.

1. A phrase is considered to contain richer and more precise information than a plain words sequence.

Example 1: Mike takes everything he **got from his** family for granted.

Example 2: Mike **takes** everything he got from his family **for granted**.

The bold text in Example 1 is just a consecutive word sequence and constitutes in no way a phrase in the linguistic sense. While in Example 2, the bold text **takes … for granted** is a linguistic phrase, though the words are separated. It is obvious that these three words would not present the full meaning unless they are interpreted as a whole. From the linguistics aspect, an intuitive hypothesis is that a phrase level representation learning can conserve the phrasal information in a more intact way and is important to the IDRR problem. Additionally, from the natural language learning aspect, several works have been conducted to conserve the phrase semantics by processing the embedding at the phrase level [24,25]. Both these articles investigated the methods of phrase embeddings. As far as I know, they were the first to apply phrase embeddings to the problem of IDRR.

The other motivation is a consequence of Motivation 1. If the words in a phrase are regarded as a whole, the syntactic structure of the sentence would become simpler.

2. A sentence with simpler syntactic constituents is easier for models to interpret.

If a phrase is regarded as a single semantic unit, the syntactic constituents of the text would be more concise.

Example 3: Mike took the weather into account.

Example 4: Mike took_into_account the weather.

The syntactic constituents of Example 3 are in the form of Subject-Verb-Object-Complement ([Mike]-[took]-[the weather]-[into account], SVOC). If we consider the phrase took … into account as a single unit, the sentence could be reconstructed as what Example 4 shows. The syntactic constituents would then be simplified to the form Subject-Verb-Object ([Mike]-[took_into_account]-[the weather], SVO). The syntactic constituents would be more simple when the words of a phrase are regarded as a unit. For example, the verb and complement in Example 3 are combined into a single word in Example 4. Less complexity of a sentence would allow the training process to focus more on its ultimate mission, the Discourse Relation Recognition.

To be brief, our method consists of 3 steps.

Step 1. Phrase extraction. This step is to recognize the phrases in the sentence.

Step 2. Phrase embedding. The words of a phrase will be embedded into a single embedding with the same dimension as other word embeddings. The syntactic constituents would consequently be simplified as well.

Step 3. Implicit discourse relation recognition. The above embeddings would be sent to an IDRR model.

We summarize the contributions in three points: (1) As far as we known, we are the first to apply phrase level embedding in the problem of IDRR and we showed that the semantic unit level representation and the simplification of the syntactic constituents help to improve the performance of the IDRR problem. (2) We proposed three different methods to segment a sentence at the level of semantic units, which can be easily implemented into existing IDRR models. Performance is compared between these three methods. (3) Our results outperform the baseline model on the F1 score by 1.7% for the corpus-based method, showing that a proper embedding level can improve the pretrained model’s performance on the IDRR problem.

## 2. Related Works

In this section, we discuss some works closely related to the three steps of the framework.

The IDRR problem is commonly studied using the Penn Discourse Treebank 2.0 (PDTB 2.0) [26]. PDTB 2.0 was released in 2008 and is used in many shallow semantic parsing problems [27,28]. The corpus is constructed from 2312 Wall Street Journal (WSJ) articles. It presents useful information about every two consecutive sentences, for example, the discourse relation, the connective of the sentence pair, if there is one, and the dependency tree of each sentence in the format of JSON, etc. Among this information, we care most about the discourse relation of the two sentences. PDTB arranges the relations into a hierarchy of three levels. The top level categorizes the relations into four classes, including contingency (Cont.), comparison (Comp.), expansion (Exp.), and temporal (Temp.). The second and third levels further categorize the above 4 classes into 14 types and 23 subtypes, respectively. The relation of the sentence pair is annotated manually and can be used as the gold standard. Researchers of the IDRR problem usually evaluate their works only on the top level. PDTB 2.0 is also the dataset we used in our work.

Step 1 in our work aims at extracting the phrases in the sentence. The commonly used methods for this task are usually based on graphs [29,30] or probabilistics [31,32]. Inspiring as they are, the results of these methods are still not satisfying enough for application; accuracy and recall are problems. Another is the phrases extracted by these methods often are not the phrases in the semantic and academic sense. The reliability of the extracted phrases is a key factor for our work, so we decided to use a less sophisticated but relatively robust method. That is, to obtain a list of frequent phrases used in daily life, phrases in sentences were extracted using a pattern matching strategy. The phrase list was collected from Wiktionary. It is a crowd-sourced dictionary that contain words, phrases, and idioms in natural languages. The terms in Wiktionary are of clearly widespread use and/or have appeared in permanently recorded media with at least three independent instances that cover at least one year. This assures that the phrases in Wiktionary would be what we actually use in daily life, not just some word sets with the form of a phrase. As to the pattern matching strategy, we wrote the pattern with Regex, along with the Python package, Pattern [33]. Ref. [33] proposed a toolkit whose different modules provide functions in web mining, natural language processing, machine learning, and network analysis, etc. We mainly use the module Pattern.en to deal with the conjugations.

Step 2 embeds a phrase into a single embedding with the same dimension of other single words. To embed a language of a morpho-phonemic writing system, e.g., English, word is the natural choice for the embedding granularity because words stand alone for a complete semantic unit in the general cases. Still, it exists ubiquitously that the integrity of a semantic unit only holds at the phrase level. For example, the phrase with respect to would lose its semantics unless these three words are comprehended as a whole. While word embedding has been widely studied and its performance proved by many NLP tasks, phrase embedding still lags. There is still no phrase embedding model as dominant as the BERT model for word embedding. Ref. [25] was inspired by the classic ideas of Skip-Gram [34], CBOW [35], and GloVe [36]. The authors put forward Phrase2Vec, which included three models for phrase embedding, namely Skip-Phrase, CBOP (Continuous Bag of Phrases), and GloVeFP (Global Vectors for Phrase Representation). Ref. [37] proposed a method based on phrase structure and context to compose phrase embeddings from word embeddings. Experiments showed improvements on language modeling and several phrase similarity tasks. Ref. [38] used an auto-encoder-based method to generate phrase embeddings from pretrained phrase embeddings and word embeddings, learning both the internal and external information of phrases. Ref. [24] proposed a contrastive objective and fine-tuned BERT (named Phrase-BERT) on a constructed phrase dataset. The produced phrase embeddings showed improvements on several phrase-level similarity tasks. In Step 2 we decided to use the pretrained Phrase-BERT to embed the phrases extracted from Step 1. Phrase-BERT and the IDRR model in Step 3 were all fine-tuned from BERT. The phrase embeddings of the pretrained Phrase-BERT should be more compatible compared to other phrase embeddings models.

Step 3 is the prediction of the discourse relation. IDRR has long been one of the most difficult problems in shallow discourse parsing. Methods range from the machine learning way to the deep learning way [39,40,41]. Ref. [39] combined the semantic interaction with the topic continuity and attribution of the two target arguments to infer the discourse relation. Patterns have been designed for the four basic classes of discourse relations, respectively. Ref. [42] used a prompt-tuning method to turn the classification problem into the mask prediction. Ref. [43] demonstrated that the Next Sentence Prediction task enhances the prediction of implicit discourse relation. Ref. [44] noticed the dependence between different levels of discourse relation label and proposed a model with a label attentive encoder. Ref. [45] adopted in their model different grained text representations, including character, subword, word, sentence, and sentence pair levels and achieved state-of-the-art accuracy and F1 scores. Ref. [46] proposed a sentence-level attention-based model for relation extraction. BMGF-RoBERTa [23] also emphasized the importance of the embedding granularity. They used word and sentence level embeddings and constructed a bilateral multi-perspective matching module along with a global information fusion module in their model. In Step 3 we used the architecture of BMGF-RoBERTa to predict the discourse relation for two reasons. First, BMGF-RoBERTa should be compatible with Phrase-BERT in Step 2. Second, to show that a properly chosen embeddings granularity and the simplification of syntactic constituents could still bring considerable improvement.

Compared to the previous works on IDRR, we chose to embed the sentence at the phrase level instead of word level, for the purpose of protecting the semantic completeness of phrases. The details of our methods will be introduced in the next section.

## 3. Methods

Here we give a brief introduction to our workflow designed out of the two motivations.

**Figure 1:**
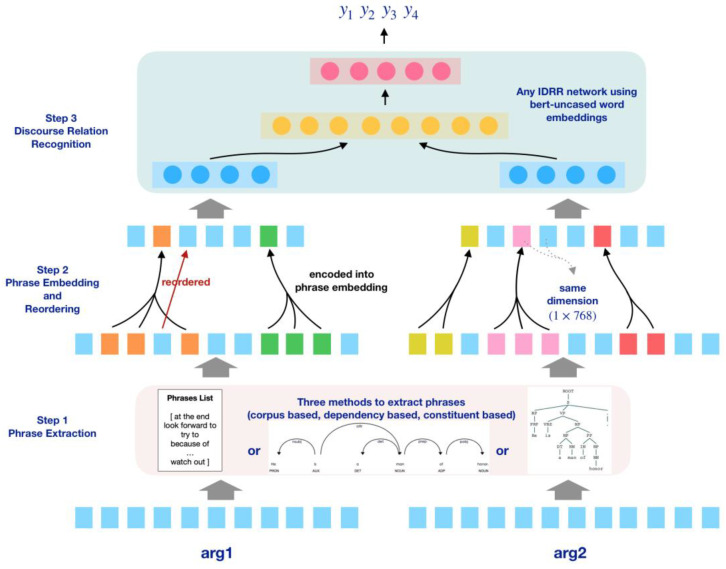
Overview of the workflow.

Figure 1 shows an overview of our workflow. Step 1, we preprocessed the corpus to extract the phrases in the sentence. Three different methods were used in this step. The first one was a corpus-based method. We gathered 5910 commonly used English phrases from Wiktionary [47] and extracted the phrases of each sentence in the corpus by a pattern matching strategy. The other two methods were based on a constituent parsing tree and a dependency parsing tree, respectively. Phrases were extracted according to the constituent structure or the dependency in the sentence. Step 2 was to embed the sentence at the level of semantic units. For the phrases extracted in step 1, we embedded each one of them (the squares with the same color constitute a phrase) into a single embedding with the same dimension (768 for BERT’s case) as other single words in the sentence, and the structure of the syntactic constituents were consequently simplified. If the phrase contained an object inside (the red arrow in Figure 1), the sentence was reordered to put the object right after the phrase. The embeddings of the single words were collected from the pretrained BERT [48] model and the phrase embeddings from the pretrained Phrase-BERT model [24]. Step 3, we engaged the training process using the architecture of BMGF-RoBERTa [23] but chose the BERT encoder instead of RoBERTa [49] because the phrase level embeddings were not compatible with the subword level embeddings of RoBERTa.

The model tries to conserve the full information of semantic units in a sentence and simplify the syntactic constituents in order to better predict the discourse relation. A key factor is to tokenize and embed a sentence by its semantic units (phrases, or words if they stand alone for a semantic unit) but not simply by words. Tokenization by semantic units would consequently lead to the simplification of syntactic constituents. The whole process of the model can be divided to three steps, phrase extraction, word and phrase embedding, and discourse relation prediction, respectively.

### 3.1. Phrase Extraction

The significance of phrase extraction to our work is to achieve proper segmentation of semantic units inside a sentence. Phrases provide useful semantic information for the IDRR problem. Machine learning methods for IDRR usually rely on lexical features [15,16] (e.g., word pair) or syntactic features [19,20] (e.g., syntactic production rules). These features can be word or phrase level. However, deep learning IDRR models seldom exploit the rich semantics at the phrase level. Although the importance of different embedding granularities has been emphasized [23,45], the importance of different embedding granularities and phrase embeddings were not used in their models.

#### 3.1.1. Corpus-Based Method

Current methods for phrase extraction have made great progress. Still, the phrases extracted by these methods are not usually those of natural language and cannot represent a correct semantic unit. Since the correctness of the extracted phrase plays an important role in our work, we decided to take a less delicate, but more assuring way. That is, to collect a list of frequent phrases and conduct a pattern matching strategy to extract phrases in the corpus. This method served as baseline and was compared to the constituent parsing tree-based and the dependency parsing tree-based methods.

In total, we gathered 5910 frequent phrases from Wiktionary. These English phrases were grouped into eight categories by Wiktionary, adverb-adjective phrases, alliterative phrases, non-constituents, phrasal prepositions, phrasal verbs, phrasebook, prepositional phrases, and rhyming phrases, respectively. We have ignored the alliterative phrases, non-constituents, phrasebook, and rhyming phrases categories, whose phrases either do not refer to a complete semantic unit or are more likely to be a sentence structure.

The number of phrases in each category is illustrated in Table 1.

Adverb-adjective phrases are collocations of adverbs and adjectives that co-occur more often than would be expected by chance, e.g., statistically significant. Phrasal prepositions refer to the locutions that function as a preposition, e.g., in regard to. Though phrasal prepositions are not numerous compared to other categories, most of them are frequent in daily expressions. A phrasal verb usually consists of a verb and an adverb or particle, conveying an idiomatic meaning not easily inferred from its individual words, e.g., come up with. Phrasal verbs are mostly bigram but trigram or more are frequent nonetheless. Prepositional phrases are phrases headed by a preposition along with its object or complement, and usually function like an adverb or an adjective, e.g., at a glance. Prepositional phrases are numerous as well as frequent in daily life.

The phrases we chose are phrases in the common sense. They are not necessarily to be grammatical phrases, but they all convey certain ideas. A common point of them is that they contain plural words but, as a whole, represent a small but complete semantic unit. Any isolated understanding of words in a phrase would lead to a loss of semantics. From this point of view, the tokenization of English sentences should be realized by semantic units, not simply by words.

Patterns were designed to extract the phrases listed above from PDTB 2.0. The 5910 phrases in the list are in their original form, but the situation is a bit more complicated in the corpus. The pattern must cover different inflections, as well as the insertion of other words inside a phrase. Phrasal prepositions and prepositional phrases are relatively simple and mostly are in the original form. Adverb-adjective phrases have different declensions. Phrasal verbs have various conjugations as well as declensions. In addition, the words in phrasal verbs are not necessarily consecutive and an object could be placed inside, just like take…into account in Example 3.

We designed a pattern with the aid of Regex and the Python package, Pattern.en. Pattern.en provides functions to deal with all inflections. We integrated all singular and plural forms of nouns, comparative and superlative forms of adjectives, and all conjugations for verbs in the pattern. In case of inserted words inside a phrasal verb, we allowed at most a trigram between the verb and the other part.

#### 3.1.2. Constituent Parsing Tree-Based Method

The constituent parsing aims at finding out the constituent structure of a sentence. Words are grouped into different phrases according to their grammatical role.

**Figure 2:**
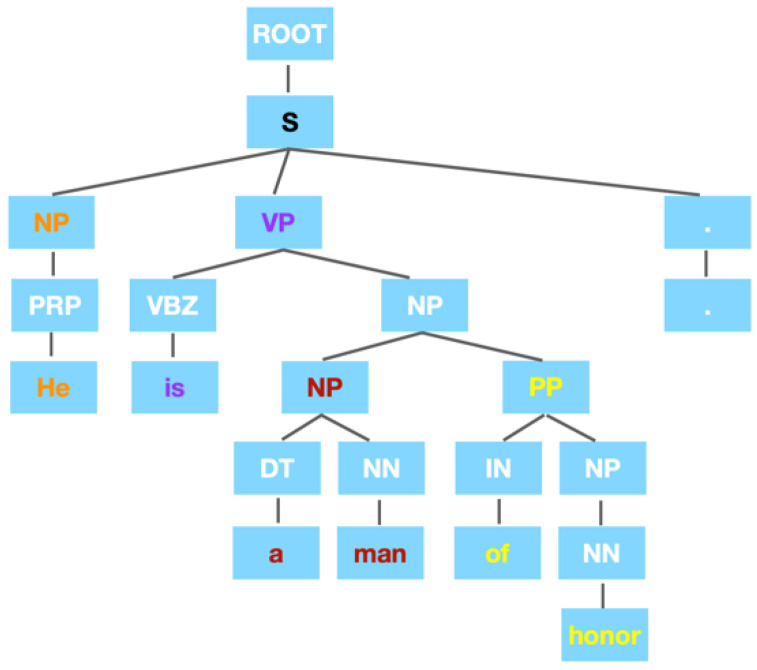
Constituent parsing tree of the sentence, he is a man of honor. The text in different color is just used to explain our method.

Figure 2 is an example of a constituent parsing tree, whose raw text is, he is a man of honor. The leaves represent the words of the sentences. The direct parent node of each leaf tells the Part-of-Speech (POS) tag of its leaf. The rest nodes represent a certain kind of phrase type such as NP (Noun Phrase), and PP (Prepositional Phrase), etc. As can be seen, the phrases provided by the constituent parsing tree are not necessarily those in the daily life. Defective as it might be, this method gives a broader view of the concept of a phrase. In comparison to the phrases collected in Section 3.1.1, phrases in the constituent parsing tree reflect more regularity in the syntactic sense and enable a more automatic way to extract phrases.

Our method consists of finding the lowest common ancestor under the level of S (sentence, in black). For example, a man should be considered as a phrase since these two words have a lowest common ancestor NP (in red). Any other word(s) added will lead to a higher common ancestor. Consider this case: if the word is added, the common ancestor would become VP (in purple), which is a higher ancestor than NP. It is also remarkable that the word he should be considered as an independent phrase although he and is have a lowest common ancestor S (in black). Only the nodes under level S (sentence) are needed. Following this idea, the example sentence should be segmented into [he] [is] [a man] [of honor] [.].

#### 3.1.3. Dependency Parsing Tree-Based Method

The dependency parsing tree focuses more on the syntactic dependency relation between nodes. The predicate of the sentence acts as the root. All other leaves could be traced back to the root through the arrows.

**Figure 3:**
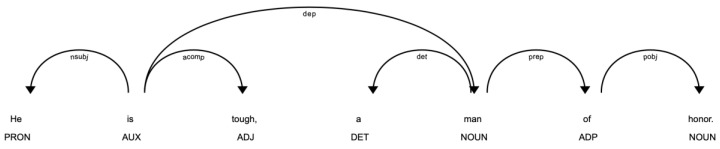
Dependency parsing tree of the sentence, he is tough, a man of honor.

Figure 3 is a dependency parsing tree made by a similar sentence to the one in Section 3.1.2, with only a slight modification in order to better explain our method. The leaves in the tree are still the words of the sentence, each one with a tag below indicating its POS. The root, or the predicate, can be located easily as no arrows would point to a root. Any other nodes would finally be related to the root through a chain of arrows. The tag under the arrow means the syntactic dependency between two nodes, such as det (determiner), and nsubj (noun subject), etc.

We segment the sentence into groups by two criteria:

1. If a leaf has no leaf siblings, we trace back through the arrows until a node with siblings or the root is met. All nodes in this chain of arrows, including the final node with siblings, are considered a phrase.

For example, the word honor has no siblings. We follow the arrow and reach the word man, which is the first node we meet with a sibling (he in this case). All these nodes we pass through are considered a phrase, that is, man of honor.

2. If a node has leaf siblings, all these nodes will be grouped to their direct parent to make a phrase.

For example, he has a leaf sibling tough, these two nodes will be grouped to their direct parent node is to make a phrase, he is tough.

We will obtain the final segmentation of [he is tough,] [a man] [man of honor.] Pseudocode could be seen in the Algorithm 1.
**Algorithm 1:** Phrase Extraction by Dependency Parsing Tree.word_sequence = [w1, w2, w3, …, wn] sequence_length = n current_word = w1 current_phrase = [ ] phrase_set = [current_phrase] while len(current_position) < sequence_length: if is_leaf(current_word) and not has_leaf_siblings(current_word) and current_word not in phrase_set: while not has_siblings(parent_of (current_word)): current_phrase = current_phrase + current_word current_word = parent_of(current_word) current_phrase = current_phrase + current_word phrase_set = phrase_set + current_phrase current_phrase = [ ] if is_leaf(current_word) and has_leaf_siblings(current_word) and current_word not in phrase_set: current_phrase = current_phrase + current_word + leaf_siblings(current_word) + parent_of(current_word) phrase_set = phrase_set + current_phrase current_phrase = [ ] current_word = next_word(current_word)

### 3.2. Phrase Extraction

Phrase extraction allow us to segment a sentence by semantic units. The next problem is how to encode these semantic units into embeddings. The pretrained BERT has already presented elegant embeddings for words. As for the Phrase Embedding, there is still no model as dominant as BERT. Ref. [25] was inspired by Word2Vec and proposed Phrase2Vec. Ref. [38] learned from internal and external phrasal information and generated phrase embeddings using both pretrained phrase embeddings and word embeddings. Ref. [24] was fine-tuned from BERT using a new contrastive objective to produce phrase embeddings. Among the studies we investigated, Phrase-BERT is the most suitable for our work. First of all, Phrase-BERT provides a pretrained phrase embedding model, enabling us to generate phrase embeddings without the time consuming pretrain process. In addition, Phrase-BERT showed good compatibility between word and phrase embeddings. The following example shows the cosine similarity between three connectives from PDTB 2.0. All these connectives are embedded by Phrase-BERT.

Example 5
in regard to = [−0.2950, 0.0351, ..., 0.0850] with respect to = [−0.3810, 0.2950, ..., −0.0822] concerning = [−0.0805, 0.1930, ..., 0.1780] CosSim(‘in regard to’, ‘with respect to’) = 0.82 CosSim(‘in regard to’, ‘concerning’) = 0.90 CosSim(‘with respect to’, ‘concerning’) = 0.71


These three expressions convey almost the same meaning and the embeddings generated by Phrase-BERT reflect the similarity between them well. In this way, phrases can conserve their semantics while being encoded with the same length as word embeddings.

In this step, we conduct an embedding of sentences by their semantic units. This requires both word embedding and phrase embedding. A sentence will first be encoded into word embeddings as any other works do, but the words that constitute a phrase would then be marked down and replaced by a single phrase embedding. As some phrases are not composed by consecutive words, e.g., take…into account, their embeddings inevitably lead to a rearrangement of words position (see Examples 3 and 4). Among the four categories of phrases we collected, phrasal verbs are the only case where words could be inserted inside. Fortunately, the inserted words can only be placed right after the verb, so after the embeddings of a phrase, all words embeddings originally following the verb could be just placed after the new phrase embedding to form a reconstructed sentence (see Examples 3 and 4).

**Figure 4:**
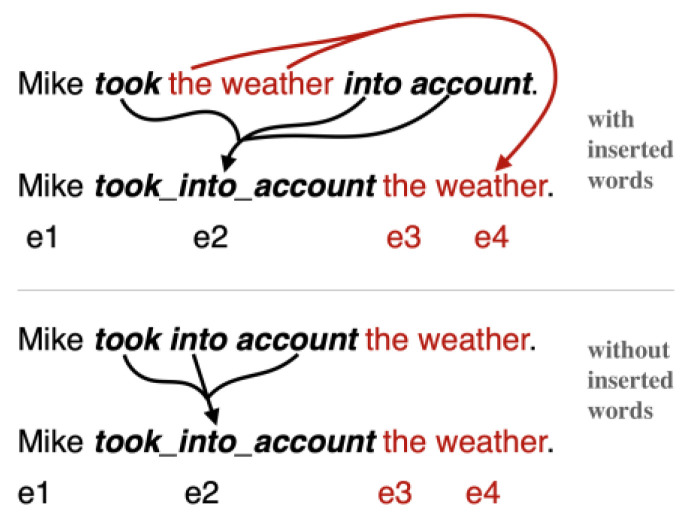
Two different cases when performing the semantic unit embeddings.

Figure 4 presents two different cases when processing the semantic unit embeddings, cases with and without inserted words, respectively. In both cases, the originally separated words of a phrase are merged into a single embedding and act as a single word (took … into account → took_into_account). The upper part of Figure 4 shows a case where some words are inserted in the phrase. The phrase embedding will unavoidably change the word position since the object (the weather) has to be put outside the phrase (took_into_account). For the case in the lower part of Figure 4, the merging of words (took, into, account) does not induce the change of word position since the object (the weather) is originally outside the phrase. All other words just keep staying where they were.

Both examples simplified the syntactic constituents as well as the dependency structure. Though mechanisms such as Self-Attention are capable of capturing the long-distance dependency between words [50], the simplification of the input sequence could nonetheless reduce the model’s distraction by the complex sentence structure and help the training process focus more on its intrinsic mission of implicit discourse relation recognition.

### 3.3. Implicit Discourse Relation Recognition

Implicit discourse relation recognition tries to predict the semantic relation between two adjacent sentences. Works have been conducted to extract the interaction of semantics between two arguments. Machine learning methods make use of some representative features such as lexical, contextual, and syntactic features. These features are designed to capture the linguistic information in the two arguments. The deep learning way, however, learns this information automatically without artificially elaborated features and currently achieves better performance than the machine learning way.

The above operations turn the input sentences into embeddings at the level of the semantic unit. In this step, we will use BMGF-RoBERTa for the IDRR task. Among all these machine learning and deep learning methods, BMGF-RoBERTa presents the best compatibility with our idea in that it also emphasizes the importance of multi-level representation of a sentence. Additionally, in the technical point of view, BMGF-RoBERTa provides encoders for the widely used pretrained models, including BERT and RoBERTa, facilitating the implementation of our semantic unit embeddings.

BMGF-RoBERTa [23] obtained its best results using the pretrained RoBERTa model, whose embedding granularity is at the word and subword level. That means the embeddings it used are not compatible with ours since our semantic units are of word and phrase level. In our work we used the pretrained BERT instead, to encode all words into embeddings and then merge certain words into phrase embeddings (Step 2). The BMGF-RoBERTa will then proceed to the bilateral multi-perspective matching and global information fusion to finally obtain the prediction.

## 4. Experiments

In this section, we give a brief look at the dataset and the experiment settings, along with a comparison of our results and the recent baselines.

### 4.1. Dataset

Our work was evaluated on the PDTB 2.0 [26], as most of the current works do. PDTB 2.0 was released in 2008 and is used in many shallow semantic parsing problems [27,28]. The corpus is constructed from 2312 Wall Street Journal (WSJ) articles. It presents useful information about every two consecutive sentences, for example the discourse relation and the connective of the sentence pair. Among this information, we care most about the discourse relation of the two sentences. PDTB 2.0 arranges the relations into a hierarchy of three levels. The top level categorizes the relations into four classes, including contingency, comparison, expansion, and temporal. The second and third levels further categorize the above 4 classes into 14 types and 23 subtypes, respectively. We evaluated our work only on the four relations of the top level because this level is sufficient for many downstream tasks. As we mostly conduct the comparison with BMGF-RoBERTa, we follow the setting of [51], as BMGF-RoBERTa did. PDTB 2.0 divides its articles into 22 sections, according to [51], Section 2–20, Section 0–1, and Section 21–22 would be used as training, validation, and test sets, respectively. Some statistics about the four top level relations can be found in Table 2 for the implicit case.

### 4.2. Parameter Settings

We conducted the evaluation for the prediction of the four relations on the top level, namely a four-way classification. Given the compatibility of embeddings, our work used the bert-base-uncased pretrained BERT instead of roberta-base by [23]. The rest settings followed [23]. Several hypermeters are set as follows. The number of perspectives of the matching layer l, the number of heads of the fusion layer h, the number of convolutional operations z, and the number of filters s were set to 16, 16, 2 and 64, respectively. Hidden dimensions were all set to 128. Dropout was applied to every layer with the rate of 0.2, The gradient L2-norm was clipped with a threshold of 2.0. The L2 regularization was performed with a coefficient of 0.0005 to avoid overfitting. We used Adam [52] as an optimizer, with an initial learning rate of 0.001 and a batch size of 32 to train models through 50 epochs.

### 4.3. Experimental Results

Our method consists of three steps, including the phrase extraction step, the phrase embedding step and the IDRR step. According to the different methods used in the phrase extraction step, we proposed three versions of model, the corpus-based version, the constituent-based version, and the dependency-based version, respectively. The experiments were conducted through the three versions and the results were compared with those of recent works.

We trained the model 3 times on 6 × 11 GB NVIDIA GeForce RTX 2080 Ti GPUs (GIGABYTE, made in Dongguan, China) and obtained the average results of the 4-way classification. Several recent works were compared with our results. Performance was evaluated by the macro-averaged F1 score and the accuracy. The main purpose of the experiment was to show the contribution of the proper embedding granularity and the simplification of syntactic constituents. While the proper embedding granularity managed to conserve the full information of semantic units, the simplification of syntactic constituents reduced the model’s distraction by the complex sentence structure and assisted the model in focusing more on the IDRR problem. Since the embedding of semantic unit intrinsically entails the simplification of sentence structure, the ablation study will not be discussed in our article.

Table 3 gives a panorama over the recent studies on the IDRR problem. Model 1 [39] used Naive Bayes as the classifier and achieved the best results among the machine learning methods so far. The rest are the deep learning methods and generally present a better performance. Models 6 and 7, two different version of BMGF-RoBERTa [23], used bert-base-uncased and roberta-base, respectively, for embeddings.

We found that the current works on IDRR face the same problem, the rich semantics inside the phrases are not fully exploited. Most works conduct the embeddings at the level of word or subword, which inevitably leads to a loss of information by splitting some words that actually belong to the same phrase.

We propose three methods (8, 9, 10) for sentence segmentation and implement them into BMGF-RoBERTa (bert-base-uncased for the reason of compatibility), trying to find a more proper embedding granularity and preserve the semantics of phrases. Results show that the corpus-based method achieved an improvement of 1.7% on F1 score in comparison to BMGF-RoBERTa (bert-base-uncased). To make full use of the rich semantics in the complete phrases, we tried to keep the completeness of the phrase during embedding; the results show that proper semantic unit segmentation and syntactic constituent simplification strategies did contribute to the implicit discourse relation recognition problem.

Two problems exist in our experiment results. First, among the three methods we proposed, the constituent-based method and the dependency-based method have not obtained better results than RoBERTa (bert-base-uncased). This might be because the semantic units obtained by these two methods are not as natural and exact as the daily phrases from the corpus. It is also a common difficulty in research of the phrase extraction problem that the phrases extracted lack semantics in a linguistic sense.

Second, though the F1 score and the F1+ accuracy is higher than the baseline model we used, the accuracy has decreased slightly. It is not surprising, because in step 2 we adopted a phrase embedding method, which would inevitably lead to some incompatibility between phrase embeddings and the BERT word embedding.

We use the following examples from PDTB 2.0 to further explain these two problems.

Example 6: “As a result of your ultimatum,” writes the Wisconsin Democrat, “I guess there is no longer any point in **taking** administration views **into account** on other items in conference, inasmuch regardless of their resolution you apparently intend to veto this bill”.

In this discourse, the three words **taking**, **into**, **account** would be encoded by Phrase-BERT into an embedding with the same dimension as the other single words (768 for BERT’s case), and then function like any other transitive verb (in this case taking into account is just like the verb-ing considering). As a result, the object inside (administration views) has to be put outside, more precisely, after the combined embedding of taking into account.

The sentence would be reorganized to: … I guess there is no longer any point in **[taking into account]** administration views on other items in conference...

This is a positive example for the phrase extraction step and the phrase embedding step that might help in classification.

The negative example is the case where we do not obtain a correct phrase:

Example 7: India’s overregulated businessmen had to be persuaded, but they have started to think big. Some of the projects being funded by the new issues are the first fruits of Mr. Gandhi’s policy, and they require more capital than the smaller industrial units **built in** the past.

In English we have the phrase **[build in]**, which means to make something part of something. However, in the above sentence, the two bold words **built in** would be mistakenly recognized as a phrase by the corpus-based phrase extraction method while **built** is actually a passive voice and does not form a phrase with the word **in.**

The above problems show us three different ways we could continue in our future work. The first thing is to find a better segmentation method in the phrase extraction step. Though we can obtain phrases from the corpus, it is, after all, time and labor consuming work. It is still necessary to design a better segmentation algorithm. Second, we should find a better phrase representation method in step 2. Third, our work is based on the baseline model BMGF-RoBERTa. In order to achieve the best performance, which we expect for our method, it is necessary to design a customized model for the IDRR, especially on the semantic unit level.

## 5. Conclusions

IDRR methods for the English language used to ignore the importance of a proper tokenization level, as word level tokenization intuitive and works on many tasks. However, isolated words usually do not stand for a full semantic unit and thus lead to a loss of information when being embedded. In attempt to make full use of the semantics of phrases for the IDRR problem, we put forward an IDRR framework, including a phrase extraction step to keep the completeness of semantic units, a phrase embedding step to conserve the semantic unit information and simplify the syntactic constituents, and finally an IDRR step to achieve the discourse relation.

Compared to the baseline model, which embeds the sentence at the word level, our results show an improvement on F1 score, showing that our strategy helps to improve the performance of IDRR models, as phrase level embeddings better conserve the semantic unit information and simplify syntactic constituents.

However, the improvement is slight. The phrases converse more complete semantic information, but on the other hand, the phrase extraction step and the phrase embedding step might lose some of the information as well. This problem also shows us the way to continue in future works; we should improve the phrase extraction and embedding algorithms.

## Figures and Tables

**Table 1:** Statistics of the phrases collected from Wiktionary.

Category	Number
Adverb-adjective Phrases	144
Phrasal Prepositions	54
Phrasal Verbs	3227
Prepositional Phrases	2474

**Table 2:** Statics of the datasets.

Datasets	Cont.	Comp.	Exp.	Temp.
Training set	3342	1942	7004	12,362
Validation set	295	197	671	64
Test set	279	152	574	85

**Table 3:** F1 score and accuracy comparison between recent models and our three methods.

Model	F1 (%)	Accuracy (%)
1. [53]	46.46	-
2. [39]	47.15	-
3. [54]	51.84	60.52
4. [55]	58.48	65.26
5. [56]	54.20	63.10
6. [23] (bert-base-uncased)	52.12	64.05
7. [23] (roberta-base)	63.29	69.06
8. Corpus-based	53.86	63.86
9. Constituent-based	28.67	55.70
10. Dependency-based	51.64	63.99

## Data Availability

https://catalog.ldc.upenn.edu/LDC2008T05 (accessed on 17 July 2023).

## References

[1] Hu C., Yang Y., Wu X. (2020). Survey of Implicit Discourse Relation Recognition Based on Deep Learning. Comput. Sci..

[2] Rutherford A.T., Demberg V., Xue N. (2017). A systematic study of neural discourse models for implicit discourse relation. Proceedings of the 15th Conference of the European Chapter of the Association for Computational Linguistics.

[3] Xue N., Ng H.T., Pradhan S., Prasad R., Bryant C., Rutherford A. (2015). The conll-2015 shared task on shallow discourse parsing. Proceedings of the Nineteenth Conference on Computational Natural Language Learning-Shared Task (CoNLL ’15).

[4] Xue N., Ng H.T., Pradhan S., Rutherford A., Webber B., Wang C., Wang H. (2016). Conll 2016 shared task on multilingual shallow discourse parsing. Proceedings of the Twentieth Conference on Computational Natural Language Learning-Shared Task (CoNLL ’16).

[5] Gerani S., Mehdad Y., Carenini G., Ng R., Nejat B. (2014). Abstractive summarization of product reviews using discourse structure. Proceedings of the 2014 Conference on Empirical Methods in Natural Language Processing (EMNLP ’14).

[6] Louis A., Joshi A.K., Nenkova A. (2010). Discourse indicators for content selection in Summarization. Proceedings of the 11th Annual Meeting of the Special Interest Group on Discourse and Dialogue (SIGDIAL ’10).

[7] Yoshida Y., Suzuki J., Hirao T., Nagata M. (2014). Dependency-based discourse parser for single-document summarization. Proceedings of the 2014 Conference on Empirical Methods in Natural Language Processing (EMNLP ’14).

[8] Jansen P., Surdeanu M., Clark P. (2014). Discourse complements lexical semantics for non-factoid answer reranking. Proceedings of the 52nd Annual Meeting of the Association for Computational Linguistics (ACL ’14).

[9] Liakata M., Dobnik S., Saha S., Batchelor C., Schuhmann D.R. (2013). A discourse-driven content model for summarising scientific articles evaluated in a complex question answering task. Proceedings of the 2013 Conference on Empirical Methods in Natural Language Processing (EMNLP ’13).

[10] Verberne S., Boves L., Oostdijk N., Coppen P.-A. (2007). Evaluating discourse-based answer extraction for why-question answering. Proceedings of the 30th Annual International ACM SIGIR Conference on Research and Development in Information Retrieval (SIGIR ’07).

[11] Shafiq M., Tian Z., Bashir A.K., Du X., Guizani M. (2020). CorrAUC: A malicious bot-iot traffic detection method in iot network using machine-learning techniques. IEEE Internet Things J..

[12] Chai Y., Du L., Qiu J., Yin L., Tian Z. (2022). Dynamic Prototype Network based on Sample Adaptation for Few-Shot Malware Detection. IEEE Trans. Knowl. Data Eng..

[13] McKeown O.B.K. (2013). Aggregated Word Pair Features for Implicit Discourse Relation Disambiguation. Proceedings of the 51st Annual Meeting of the Association for Computational Linguistics (ACL’13).

[14] Blair-Goldensohn S., McKeown K., Rambow O. (2007). Building and Refining Rhetorical-Semantic Relation Models. Human Language Technologies 2007: The Conference of the North American Chapter of the Association for Computational Linguistics, Proceedings of the Main Conference (NAACL ’07), Rochester, NY, USA, 22–27 April 2007.

[15] Pitler E., Louis A., Nenkova A. (2009). Automatic sense prediction for implicit discourse relations in text. Proceedings of the 47th Annual Meeting of the Association for Computational Linguistics and the 4th International Joint Conference on Natural Language Processing of the AFNLP (ACL ’09).

[16] Pitler E., Raghupathy M., Mehta H., Nenkova A., Lee A., Joshi A.K. (2008). Easily Identifiable Discourse Relations. Proceedings of the 22nd International Conference on Computational Linguistics, Posters Proceedings (COLING ’08).

[17] Li H., Zhang J., Zong C. (2015). Predicting Implicit Discourse Relations with Purely Distributed Representations. Proceedings of the Chinese Computational Linguistics and Natural Language Processing Based on Naturally Annotated Big Data.

[18] Li J.J., Nenkova A. (2014). Reducing sparsity improves the recognition of implicit discourse relations. Proceedings of the 15th Annual Meeting of the Special Interest Group on Discourse and Dialogue (SIGDIAL’14).

[19] Zhou Z.M., Lan M., Niu Z.-Y., Xu Y., Su J. (2010). The effects of discourse connectives prediction on implicit discourse relation recognition. Proceedings of the SIGDIAL 2010 Conference (SIGDIAL ’10).

[20] Zhou Z.-M., Xu Y., Niu Z.-Y., Lan M., Su J., Tan C.L. (2010). Predicting discourse connectives for implicit discourse relation recognition. Proceedings of the 23rd International Conference on Computational Linguistics: Posters (COLING ’10).

[21] Shafiq M., Tian Z., Bashir A.K., Du X., Guizani M. (2020). IoT malicious traffic identification using wrapper-based feature selection mechanisms. Comput. Secur..

[22] Chai Y., Qiu J., Yin L., Zhang L., Gupta B.B., Tian Z. (2022). From Data and Model Levels: Improve the Performance of Few-Shot Malware Classification. IEEE Trans. Netw. Serv. Manag..

[23] Liu X., Ou J., Song Y., Jiang X. (2020). On the Importance of Word and Sentence Representation Learning in Implicit Discourse Relation Classification. Proceedings of the Twenty-Ninth International Joint Conference on Artificial Intelligence (IJCAI’20).

[24] Wang S., Thompson L., Iyyer M. Phrase-BERT: Improved Phrase Embeddings from BERT with an Application to Corpus Exploration. Proceedings of the 2021 Conference on Empirical Methods in Natural Language Processing.

[25] Wu Y., Zhao S., Li W. (2019). Phrase2Vec: Phrase embedding based on parsing. Inf. Sci..

[26] Prasad R., Dinesh N., Lee A., Miltsakaki E., Robaldo L., Joshi A.K., Webber B.L. (2008). The Penn Discourse TreeBank 2.0. Proceedings of the International Conference on Language Resources and Evaluation (LREC ’08).

[27] Bosselut A., Celikyilmaz A., He X., Gao J., Huang P.S., Choi Y. Discourse-aware neural rewards for coherent text generation. Proceedings of the NAACL-HLT.

[28] Cohan A., Dernoncourt F., Kim D.S., Bui T., Kim S., Chang W., Goharian N. A discourse-aware attention model for abstractive summarization of long documents. Proceedings of the NAACL-HLT.

[29] Gebhardt K., Nederhof M.-J., Vogler H. (2017). Hybrid Grammars for Parsing of Discontinuous Phrase Structures and Non-Projective Dependency Structures. Comput. Linguist..

[30] Jie Z., Muis A., Lu W. Efficient dependency-guided named entity recognition. Proceedings of the Thirty-First AAAI Conference on Artificial Intelligence.

[31] Liu J., Shang J., Wang C., Ren X., Han J. Mining quality phrases from massive text corpora. Proceedings of the 2015 ACM SIGMOD International Conference on Management of Data, ACM.

[32] Sánchez-Cartagena V.M., Pérez-Ortiz J.A., Sánchez-Martínez F. (2016). Integrating rules and dictionaries from shallow-transfer machine translation into phrase-based statistical machine translation. J. Artif. Intell. Res..

[33] De Smedt T., Daelemans W. (2012). Pattern for python. J. Mach. Learn. Res..

[34] Mikolov T., Chen K., Corrado G., Dean J. (2013). Efficient estimation of word representations in vector space. arXiv.

[35] Mikolov T., Sutskever I., Chen K., Corrado G.S., Dean J. (2013). Distributed representations of words and phrases and their compositionality. Advances in Neural Information Processing Systems.

[36] Pennington J., Socher R., Manning C.D. Glove: Global vectors for word representation. Proceedings of the 2014 Conference on Empirical Methods in Natural Language Processing (EMNLP ’14).

[37] Yu M., Dredze M. (2015). Learning composition models for phrase embeddings. Trans. Assoc. Comput. Linguist..

[38] Li R., Yu Q., Huang S., Shen L., Wei C., Sun X. (2020). Phrase embedding learning from internal and external information based on autoencoder. Inf. Process. Manag..

[39] Lei W., Xiang Y., Wang Y., Zhong Q., Liu M., Kan M.Y. Linguistic properties matter for implicit discourse relation recognition: Combining semantic interaction, topic continuity and attribution. Proceedings of the AAAI Conference on Artificial Intelligence.

[40] Zhang N., Deng S., Sun Z., Wang G., Chen X., Zhang W., Chen H. (2019). Long-tail relation extraction via knowledge graph embeddings and graph convolution networks. arXiv.

[41] Gao T., Han X., Liu Z., Sun M. Hybrid attention-based prototypical networks for noisy few-shot relation classification. Proceedings of the AAAI Conference on Artificial Intelligence.

[42] Chen X., Zhang N., Xie X., Deng S., Yao Y., Tan C., Huang F., Si L., Chen H. Knowprompt: Knowledge-aware prompt-tuning with synergistic optimization for relation extraction. Proceedings of the ACM Web Conference.

[43] Wei S., Demberg V. Next sentence prediction helps implicit discourse relation classification within and across domains. Proceedings of the 2019 Conference on Empirical Methods in Natural Language Processing and the 9th International Joint Conference on Natural Language Processing (EMNLP-IJCNLP).

[44] Wu C., Cao L., Ge Y., Liu Y., Zhang M., Su J. A Label Dependence-aware Sequence Generation Model for Multi-level Implicit Discourse Relation Recognition. Proceedings of the AAAI Conference on Artificial Intelligence.

[45] Bai H., Zhao H. Deep Enhanced Representation for Implicit Discourse Relation Recognition. Proceedings of the 27th International Conference on Computational Linguistics.

[46] Lin Y., Shen S., Liu Z., Luan H., Sun M. Neural relation extraction with selective attention over instance. Proceedings of the 54th Annual Meeting of the Association for Computational Linguistics.

[47] Jimmy Wales Wikimedia Community. https://en.wiktionary.org/wiki/Wiktionary:Main_Page.

[48] Devlin J., Chang M.-W., Lee K., Toutanova K. (2019). BERT: Pre-training of Deep Bidirectional Transformers for Language Understanding. Proceedings of the 2019 Conference of the North American Chapter of the Association for Computational Linguistics: Human Language Technologies (NAACL ’19).

[49] Liu Y., Ott M., Goyal N., Du J., Joshi M., Chen D., Levy O., Lewis M., Zettlemoyer L., Stoyanov V. (2019). Roberta: A robustly optimized bert pretraining approach. arXiv.

[50] Ashish Vaswani N., Parmar N., Uszkoreit J., Jones L., Gomez A.N., Kaiser Ł., Polosukhin I. (2017). Attention is all you need. Advances in Neural Information Processing Systems.

[51] Ji Y., Eisenstein J. (2015). One Vector is Not Enough: Entity-Augmented Distributed Semantics for Discourse Relations. Trans. Assoc. Comput. Linguist..

[52] Kingma D.P., Ba J. Adam: A method for stochastic optimization. Proceedings of the 3rd International Conference on Learning Representations, ICLR.

[53] Lei W., Wang X., Liu M., Ilievski I., He X., Kan M.-Y. (2017). SWIM: A simple word interaction model for implicit discourse relation recognition. Proceedings of the 26th International Joint Conference on Artificial Intelligence (IJCAI ’17).

[54] Varia S., Hidey C., Chakrabarty T. (2019). Discourse Relation Prediction: Revisiting Word Pairs with Convolutional Networks. Proceedings of the 20th Annual SIGdial Meeting on Discourse and Dialogue (SIGDIAL ’19).

[55] Kishimoto Y., Murawaki Y., Kurohashi S. (2020). Adapting BERT to Implicit Discourse Relation Classification with a Focus on Discourse Connectives. Proceedings of the 12th Language Resources and Evaluation Conference (LREC ’20).

[56] Munir K., Zhao H., Li Z. (2021). Learning Context-Aware Convolutional Filters for Implicit Discourse Relation Classification. IEEE/ACM Trans. Audio Speech Lang. Process..

